# Usefulness of Lateral Lumbar Interbody Fusion Combined with Indirect Decompression for Degenerative Lumbar Spondylolisthesis: A Systematic Review

**DOI:** 10.3390/medicina58040492

**Published:** 2022-03-29

**Authors:** Takuya Nikaido, Shin-ichi Konno

**Affiliations:** Department of Orthopaedic Surgery, Fukushima Medical University School of Medicine, 1 Hikari-gaoka, Fukushima City 960-1295, Japan; skonno@fmu.ac.jp

**Keywords:** degenerative lumbar spondylolisthesis (DS), minimally invasive spine surgery (MISS), minimally invasive spinal stabilization (MISt), lateral lumbar interbody fusion (LLIF), percutaneous pedicle screw (PPS), indirect decompression

## Abstract

*Background and Objective:* The aim of this review was to analyze the existing literature and investigate the outcomes or complications of lateral lumbar interbody fusion (LLIF) combined with indirect decompression for degenerative lumbar spondylolisthesis (DS). *Materials and Methods:* A database search algorithm was used to query MEDLINE, COCHRANE, and EMBASE to identify the literature reporting LLIF with indirect decompression for DS between January 2010 and December 2021. Improvements in outcome measures and complication rates were pooled and tested for significance. *Results:* A total of 412 publications were assessed, and 12 studies satisfied the inclusion criteria after full review. The pooled data available in the included studies showed that 438 patients with lumbar spondylolisthesis (mean age 65.2 years; mean body mass index (BMI) 38.1 kg/m^2^) underwent LLIF. A total of 546 disc spaces were operated on. The most frequently treated levels were L4–L5 and L3–L4. Clinically, the average improvement was 32.5% in ODI, 46.3 mm in low back pain, and 48.3 mm in leg pain estimated from the studies included. SF-36 PCS improved by 51.5% and MCS improved by 19.5%. For radiological outcomes, a reduction in slippage was seen in 6.3%. Disc height increased by 55%, foraminal height increased by 21.1%, the foraminal area on the approach side increased by 21.9%, and on the opposite side it increased by 26.1%. The cross-sectional spinal canal area increased by 20.6% after surgery. Post-operative complications occurred in 5–40% of patients with thigh symptoms, such as anterior thigh numbness, dysesthesia, discomfort, pain, and sensory deficits. *Conclusions:* Indirect decompression by LLIF for DS is an effective method for improving pain and dysfunction with less surgical invasion. In addition, it has the effect of significantly improving disc height, foraminal height and area, and segmental lordosis on radiological outcomes compared to the posterior approach.

## 1. Introduction

With the recent needs of patients in a super-aging society for healthy longevity, minimally invasive surgery (MIS) is attracting attention in the field of spinal surgery. Due to the aging population and the needs of patients with spinal disorders, a variety of MIS techniques are gaining popularity. In particular, procedures using a percutaneous pedicle screw (PPS), such as MIS-transforaminal lumbar interbody fusion (TLIF) and MIS long-fixation, are widely practiced [1,2,3,4]. Lumbar interbody fusion (LIF) has spread as a minimally invasive spine surgery (MISS) since its first report in the United States in 2006 [5], and the eXtreme Lumbar Interbody Fusion (XLIF) and the Oblique Lumbar Interbody Fusion (OLIF) are useful devices. Due to the high quality of these devices, they are now used globally. LIF can provide interbody correction for spinal deformity and indirect decompression of spinal stenosis [6]. In addition, for LIF combinations with posterior fixation, percutaneous reduction with a combination of LIF and PPS (LIF/PPS) is becoming the standard procedure for reducing invasiveness and maintaining the back muscles. For lumbar fusions resulting from degenerative spinal disorders such as degenerative spondylolisthesis (DS), LIF/PPS is expected to achieve clinical outcomes equal to or improved to MIS-TLIF [7,8,9]. Indirect decompression, in particular, is a method of indirectly expanding the spinal canal by correcting the disc height and slip that have decreased due to degeneration. The spinal canal can be decompressed without any operation, and by LIF/PPS, it is possible to perform posterior fixation without deploying an initial surgical wound. However, factors that contribute to indirect decompression include patient factors such as ligamentum flavum width and protruded disc, pre-operative dural sac area, facet joint degeneration, pre-operative disc height, and implant factors such as cage subsidence and cage height. Many surgery-related factors such as these have been reported, and the usefulness and limitations of indirect decompression have not been clarified. Furthermore, many of the reports so far have performed evaluations of spinal stenosis and spinal deformity cases, including various pathologies, and the effects of LIF and indirect decompression for lumbar degenerative spondylolisthesis are unknown [10,11,12,13]. The purpose of this review was to analyze the existing literature and investigate the outcomes or complications of LLIF combined with indirect decompression and additional direct posterior decompression for lumbar degenerative spondylolisthesis.

## 2. Materials and Methods

Literature Search Strategy: Medline through PubMed, Embase, and the Cochrane Central Register of Controlled Trials were searched from the earliest available date of indexing between January 2010 and December 2021. The search strategy was based on the following title/abstract key words/MeSH terms: “spondylolisthesis”, AND “indirect decompression” AND “lateral lumbar interbody fusion” OR “oblique lumbar interbody fusion” OR “extreme lateral interbody fusion” OR “LLIF” OR “OLIF” OR “XLIF” OR “DLIF” OR “ELIF”. Excluded from the query results were all non-human studies, cadaveric studies, and non-English literature.

Study Selection: The inclusion criteria were retrospective or prospective studies, including randomized, controlled trials, non-randomized trials, cohort studies, case-control studies, and case series providing clinical and radiological results of LLIF with indirect decompression in spondylolisthesis. Duplicate publications were excluded, as were publications such as review articles, case reports, and letters, which do not contain original data. Articles eligible for further review were identified by performing an initial screening of the identified titles and abstracts. The second screening was based on full-text review. Two investigators (T.N. and K.W.) independently assessed the full text for eligibility; discrepancies were resolved via consensus or were determined by a third investigator (S.K.).

Data Extraction: The following information, when available, was extracted from each study: number of patients, number of treated levels, mean age of the population (years), body mass index (BMI), smoking history, diabetes history, data on surgical strategy (stand-alone LLIF, and LLIF plus posterior instrumentation), duration of surgery (min), blood loss (mL), hospital stay (days), radiological parameters (Meyerding classification, slip rate, disc height (DH), foraminal height (FH), foraminal area (FA), central canal area (CA), disc angle (DA), lumbar lordosis (LL)), clinical results (Oswestry Disability Index (ODI), Short Form-12/36 (SF-12/SF-36), and visual analogue scale of back and leg pain, as well as complications.

Risk of Bias Assessment: The Newcastle−Ottawa Scale (NOS) was used to assess the methodological quality of all publications included in this review. The scale is composed of nine items that cover three aspects: (1) subject selection (four items), (2) comparability of the two study arms (two items), and (3) outcome assessment (three items). The total scores ranged from 0 to 9, with higher scores indicating higher quality.

Synthesis of Results and Analysis: Categorical variables are expressed as numbers of cases or percentages. Continuous variables are reported as means ± standard deviation (SD).

## 3. Results

**Figure 1:**
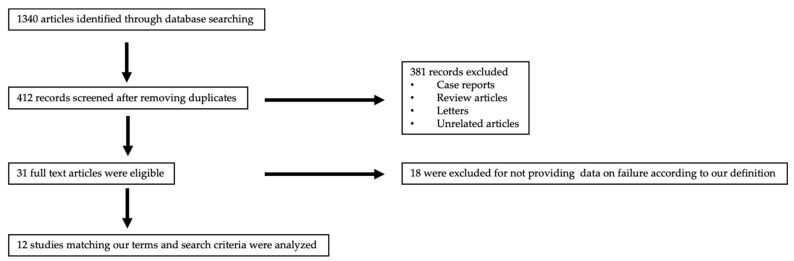
Workflow for identifying included references.

A total of 412 publications were assessed for inclusion at the title and abstract level after excluding 928 duplicate publications (Figure 1). Following the exclusion of case reports, reviews, letters, and other unrelated articles, 31 publications were eligible. Overall, 12 studies satisfied the inclusion criteria after full review. The characteristics and demographics of the included studies are presented in Table 1. The pooled data available in the included studies showed that 438 patients with lumbar spondylolisthesis (mean age 65.2 years; mean BMI 38.1 kg/m^2^) underwent LLIF. The population analyzed appeared to be uniform with respect to the available demographic data: male/female ratio and mean age; 30.9% patients were smokers and 22.7% had diabetes mellitus. The average follow-up period was 17.6 months. Table 1 shows a summary of demographic data extracted from the studies included.

### 3.1. Clinical Results

Table 2 shows a summary of the available surgical data extracted from the studies included. A total of 546 disc spaces were operated on. The most frequently treated levels were L4–L5 and L3–L4. Almost all interventions were done in the lumbar region. Slip was categorized as Grade I or II based on the Meyerding classification. The proportion of grade I cases was high. There were 10 studies of the devices, 8 of which were XLIF and 2 of which were OLIF. Posterior decompression was also used in 26 patients in only one study, but all had indirect decompression in nine studies. Twelve authors selected the standalone option (without posterior fixation) in 52 cases and combined with posterior fixation in 486 cases. In the assessment of surgery invasiveness, the mean duration of surgery was 162.5 ± 59.8 min, the mean blood loss was 139.9 ± 138.0 mL, and the mean hospitalization period was 4.5 ± 5.7 days. The authors reported clinical status on various outcome scales (Table 3 and Table 4). The most common were the VAS and ODI scores for back and leg pain. Pain intensity was assessed using a VAS in eight studies, with an average improvement of 46.3 points for low back pain and 48.3 points for leg pain. The average improvement in ODI estimated from the studies included was 32.5%, better than the minimum reported clinically significant ODI difference (11%) after spinal malformation surgery in adults. One author reported the RDQ score. RDQ improved 5.7 points from 13.9 to 8.2. SF-36 PCS improved 51.5%, from 34.5 to 52.5, and MCS improved 19.5%, from 47.4 to 56.6. SF-12 PCS improved 7.9 points, and MCS improved 6.1 points.

### 3.2. Radiological Results

Table 5 summarizes the clinical outcomes reported by the studies included. Radiological results were analyzed by nine authors based on simple radiographs, computed tomography (CT) scans, and magnetic resonance imaging (MRI). The six authors described an average reduction of slippage of 6.3%. Increased disc height (DH) from pre-operative to post-operative was reported by seven authors, with an average increase of 55% (4.0 mm; 95% CI, 3.2–4.5 mm). The four authors described an average improvement in the disc angle (DA) of 2.8 degrees (27.5% of the average increase). An increase in lumbar lordosis (LL) from pre-operative to post-operative was reported by five authors, with an average increase of 2.8 degrees. In the three-case series, the foraminal height (FH) was examined. FH increased by 21.1% (16−27.8%) from pre-operative to post-operative. The foraminal area on the approach side increased by 21.9%, and the opposite side increased by 26.1%. The cross-sectional spinal canal area (CSA) was investigated in the three-case series, with a 20.6% increase after surgery.

### 3.3. Post-Operative Complications

Post-operative complications occurred in 5–40% of patients, with thigh symptoms such as anterior thigh numbness, dysesthesia, discomfort, pain, and sensory deficits (Table 6). In addition, mild hip flexion weakness or psoas weakness, probably due to muscle trauma after passage through the psoas major muscle, occurred in 3.3–31%. Almost all symptoms improved during the follow-up period, and there were no long-term sequelae.

## 4. Discussion

### 4.1. Usefulness of Lif/Pps and the Strong Point of This Study

LIF has a wide range of indications, including lumbar disc disease, recurrent lumbar disc hernia, lumbar degenerative spondylolisthesis, lumbar degenerative scoliosis, reoperation after PLIF/TLIF, instability after laminectomy, and adjacent segmental lesions. There are many reports of the surgical effects of LIF on lumbar degenerative diseases, and its effectiveness has been clarified. Recently, attention has been focused on the indirect decompression effect of LIF, which has been used to treat both foraminal stenosis and mild central stenosis secondary to various degenerative spinal lesions. In LIF, the neural element is indirectly decompressed by increasing the height of the disc, the foraminal region, and the central canal region after placement of the interbody cage. On the other hand, the indications and limitations of MIS-TLIF and LIF/PPS procedures, such as direct and indirect decompression, remain unclear. In particular, there are few reports of LIF and indirect decompression limited to lumbar degenerative spondylolisthesis. This study is significant in that it systematically reviewed papers on LIF for lumbar degenerative spondylolisthesis and summarized the results.

The results of this study showed that LIF/PPS for Grade I and II lumbar degenerative spondylolisthesis improved low back pain and leg pain by about 50% without direct posterior decompression. Furthermore, it was found that ODI, which is a measure specific to low back pain disease, was improved by 57%; the physical component of SF-36, which is a health-related quality of life measure, was improved by 52%; and the psychological component was improved by 20%. These results are comparable to those reported for PLIF/TLIF with direct decompression. Moreover, in the assessment of surgical invasiveness, the duration of surgery, blood loss, and hospitalization period were equal to previous reports of PLIF/TLIF for degenerative spondylolisthesis. In pooling the data of radiological parameters, the mean DH recovery was 4.0 ± 1.7 mm (55%) and the mean DA recovery was 2.8 ± 2.5 (27.5%). Moreover, there was a mean reduction of slippage of 6.3% and a mean increase of CSA of 20.6%. LIF seems to be an efficient technique for indirect decompression according to the improvement of DH and segmental alignment. FH and FA are the most commonly evaluated parameters for assessing foraminal decompression. A mean FH increase of 21.2% was reported. The mean increase of FA was 21.9% on the approach side versus 26.1% on the contralateral side. LLIF seems to be an efficient technique for indirect foramen decompression according to radiological parameters (FH and FA).

Five of the twelve articles assessed in the systematic review were studies comparing the clinical results of LIF and PLIF/TLIF. Kono et al. reported that there were no differences in duration of surgery, hospital stay, or JOABPEQ, but XLIF had significantly less blood loss [17]. Ohba et al. observed that XLIF is advantageous in blood loss and damage to muscles, so that the patient returns to daily life quickly, and the incidence of low back pain is low [19]. Sembrano et al. demonstrated that XLIF, which is an indirect decompression technique, improved low back pain by 73% and leg pain by 79%, and MIS-TLIF, which is a direct decompression technique, improved low back pain by 64% and leg pain by 74%. Furthermore, ODI improved by 53% in XLIF and 57% in MIS-TLIF, showing the same tendency [8]. Similarly, Isaacs et al. reported that the cross-sectional spinal canal area was more improved with MIS-TLIF, whereas the height and area of the intervertebral foramen were improved more with XLIF. In other words, the characteristics of each technique appeared in the improvement items of the radiological outcomes [8]. Pawar et al. reported that LIF significantly improved disc height, FH, segmental lordosis, and lumbar lordosis compared to PLIF, but had similar clinical results for the VAS, ODI, and SF-12 [20].

Clinical results of LLIF and PLIF/TLIF have been reported in addition to the papers included in this systematic review. Du et al. compared the clinical results and imaging findings of OLIF and TLIF, with OLIF shortening the duration of surgery; reducing blood loss; shortening hospital stay; and improving disc height, FH, and segmental lordosis. On the other hand, there were no significant differences in VAS, ODI, slip reduction, and bone union rate [24]. Chang et al. reported a systematic review of OLIF and TLIF for lumbar degenerative spondylolisthesis that showed that OLIF was superior in blood loss and length of hospital stay, but comparable improvements in low back pain, leg pain, and disc height were seen [25]. Goyal et al. reviewed 308 studies, with eight studies on the surgical effect of LIF on low-grade lumbar spondylolisthesis, resulting in 47–67.5% slip correction, and an improved ODI of 38.6–54.5% was obtained [26]. In addition, no dural injury was observed, suggesting that complications in posterior surgery may be avoided.

### 4.2. Indications and Limitations of Lif for Lumbar Spondylolisthesis

LIF indications include lumbar disc disease, recurrent lumbar disc hernia, lumbar degenerative spondylolisthesis, lumbar degenerative scoliosis, and adjacent segmental disease. In principle, grade I of the Meyerding classification is indicated for lumbar degenerative spondylolisthesis, but grade II with intervertebral instability is also an indication. Indirect decompression using LIF has great advantages for reoperation cases, such as reoperation after PLIF/TLIF and intervertebral instability after laminectomy. In other words, indirect decompression, which can indirectly decompress the spinal canal without treating the scar tissue, corrects the disc height and slip that decreased due to degeneration, and by correcting the protrusion of the intervertebral disc and the deflection of the posterior longitudinal ligament and the ligamentum flavum. It can be repositioned, and the spinal canal can be enlarged. As the disc is operated from the lateral side, it is possible to reduce disc height without touching the scar tissue and nerves, and when combined with the PPS, it is possible to reduce the initial surgical wound. The ability to complete fixation without exposing the initial surgical site is also advantageous for reoperation cases. On the other hand, (1) is the scar tissue in the re-surgery case indirectly stretched by LIF to obtain a sufficient decompression effect, and (2) is there a new neuropathy due to the stretching of the adhered nerve tissue? There were concerns such as these, and it was necessary to collect evidence on its use. Ishii et al. classified 185 patients who underwent lumbar fusion for lumbar degenerative spondylolisthesis into a “recovery” or “no-recovery” group according to their improvements in symptoms [27]. Pre-operative computed tomography (CT) images were evaluated for the position of the superior articular processes at the slipping level, followed by a graded classification (grades 0–3) using the impingement line (I line), a new radiographic indicator. In DS cases that are classified as grade 2 or greater, the risk of aggravated bony lateral recess stenosis due to corrective surgery is high; therefore, indirect decompression by LIF/PPS is, in principle, contraindicated. It is an excellent index that can be evaluated. The limitation of this study is that all the data could not be integrated, because the clinical outcomes and radiographic outcomes of each study adopted in the systematic review were not standardized.

## 5. Conclusions

Indirect decompression by LIF + PPS for lumbar degenerative spondylolisthesis is an effective method for improving pain and dysfunction with less surgical invasion. In addition, it has the effect of significantly improving disc height, FH, and FA, and segmental lordosis as radiological outcomes compared to the posterior approach. On the other hand, post-operative complications occurred in 3.3–31% of patients with temporary thigh symptoms. Therefore, it is necessary to know the limitations of LIF + PPS for lumbar degenerative spondylolisthesis and to consider its indications.

## Figures and Tables

**Table 1:** Demographic data extracted from the included studies.

Study	Subjects	Mean Age (Years)	Female (%)	Mean FU Period (Months)	Mean BMI (kg/m^2^)	Smoker (%)	Diabetes Mellitus (%)
Ahmadian et al., 2013	31	61.5	71	18.2	-	-	-
Campbell et al., 2018	18	64 (10.5)	61	6.2 (2.7)	34 (7)	-	-
Isaacs et al., 2016	29	63	55	24	30.1	-	28
Khajavi et al., 2015	60	67.7 {52–86}	75	20.3	29.1 (4.8)	40	23
Kono et al., 2018	20	69.9 (7.5)	50	12	-	-	-
Marchi et al., 2012	52	67.6 (10.0)	73.1	24	27.4 (3.3)	-	-
Ohba et al., 2017	46	71.3 (8.6)	67.4	24	23.4 (4.1)	8.7	-
Pawar et al., 2015	39	59.0	79.5	16.1	29.6	-	-
Rodgers et al., 2012	63	66.4 {25–88}	84.1	12	30.8 {16.9–48.4}	74.6	14.3
Sato et al., 2017	20	69 (7.8) {55–82}	55	12 (6–24)	-	10	20
Sembrano et al., 2016	29	63	55	24	30.1	21	28
Wu et al., 2019	31	60 (9.3) {43–78}	71	18.0 (2.87) [14,15,16,17,18,19,20,21,22,23,24,25,26,27]	-	-	-
Total	438	65.2	66.4	17.6	38.1	30.9	22.7

FU—follow up; BMI—body mass index; ( )—SD; { }—range.

**Table 2:** Available surgical data extracted from the included studies.

Study	No. of Levels Treated	Fused Level	Meyerding Classification	Surgical Procedure	Posterior Decompression	Posterior Instrumentation	Duration of Surgery (Minutes)	Blood Loss (mL)	Hospital Stay (Days)
Ahmadian et al., 2013	31	L4/5: 31 (100%)	Grade I: 26 (83.9%) Grade II: 5 (16.1%)	XLIF	Indirect	Bilateral PS 100%	-	94. (61.9)	3.5
Campbell et al., 2018	20	L3/4 and L4/5: 2 (11%) L4/5: 16 (89%)	Grade I: 15 (83%) Grade II: 3 (17%)	transpsoas approach	Indirect	Bilateral PS 89% Unilateral PS 11%	165 (58)	113 (79)	-
Isaacs et al., 2016	36 1 level: 22 2 level: 7	L3/4: 12 (41%) L4/5: 24 (83%)	Grade I: 28 (97%) Grade II: 1 (3%)	XLIF	Indirect	Bilateral PS 100%	-	-	-
Khajavi et al., 2015	71 1 level: 49 (82%) 2 level: 11 (18%)	L2/3: 2 (3.3%) L3/4: 18 (30%) L4/5: 50 (83%)	Grade I: 47 (78%) Grade II: 13 (22%)	-	Indirect: 34 (56.7%)	PPS 57 95%	206 (65–426)	83 (10–1000)	1.3 (0–4)
Kono et al., 2018	20	L3/4: 6 (30%) L4/5:14 (70%)	-	XLIF	Indirect	Bilateral PS 100%	131 (23.2)	36.1 (15.3)	14.6 (7.5)
Marchi et al., 2012	52	L1–2: 2 (3.8%) L2–3: 9 (17.3%) L3–4: 14 (26.9%) L4–5: 27 (51.9%)	-	XLIF	Indirect	0% (stand-alone)	73.2 (31.4)	<50	-
Ohba et al., 2017	86	-	Grade I or Grade II	XLIF	Indirect	Bilateral PS 100%	-	51 (41)	-
Pawar et al., 2015	48	L3/4: 18 (37.5%) L4/5: 30 (62.5%) (included multilevel 9)	-	XLIF or COUGAR-SYSTEM	-	Bilateral or Unilateral PS 100%	260.2	438	-
Rodgers et al., 2012	80 1 level: 49 (77.8%) 2 level: 11 (17.5%) 3 level: 3 (4.8%)	L2–3: 2 (3.2%) L3–4: 15 (23.8%) L4–5: 61 (96.8%) L5–S1 (AxiaLIF): 2 (3.2%)	Grade II: 63 (100%)	XLIF	Indirect	Bilateral PS: 9 (14.3%) Unilateral PS: 53 (84.1%) Transpedicular facet fixation: 1 (1.6%) Internally fixated implant: 10 (15.9%)	-	-	1.2 (0–4)
Sato et al., 2017	20	L3–4 or L4–5	-	OLIF	Indirect	Bilateral PS 100%	-	-	-
Sembrano et al., 2016	36 1 level: 22 2 level: 7	L3/4: 12 (41%) L4/5: 24 (83%)	-	XLIF	Indirect	Bilateral PS 100%	171 {90–332}	<100 mL: 79%	2 (0–6)
Wu et al., 2019	46 1 level: 26 (83.9%) 2 level: 5 (16.1%)	-	Grade I: 31 (100%)	OLIF	-	-	131.3 (14.6)	163.6 (63.9)	-

XLIF—extreme lumbar interbody fusion (NuVasive Inc., San Diego, CA, USA), NR COUGAR-SYSTEM; COUGAR-DePuy Spine Inc., Raynham, MA, USA); OLIF—oblique lumbar interbody fusion (Clydesdale Spinal System: Medtronic, Mineapolis, MN); PS—pedicle screw; ( )—SD; { }—range.

**Table 3:** Patient-reported outcomes measures (pain intensity) by the included studies.

Study	VAS Back Pain Pre-op	VAS Back Pain Post-op	Diff	VAS Leg Pain Pre-op	VAS Leg Pain Post-op	Diff
Ahmadian et al., 2013	69.9 (15.1)	38.7 (30)	31.2	-	-	-
Khajavi et al., 2015	80	23	57	77	27	50
Marchi et al., 2012	78	31	47	54	23	31
Ohba et al., 2017	49 (32)	15 (26)	34	-	-	-
Pawar et al., 2015	-	-	46.3	-	-	-
Rodgers et al., 2012	87 (13)	22 (20)	65	-	-	-
Sato et al., 2017	55 (19)	19 (9)	36	81 (33)	20 (7)	61
Sembrano et al., 2016	73	19	54	70	19	51
Total	70.3 (13.7)	24.0 (8.2)	46.3 (12.0)	70.5 (11.9)	22.3 (3.6)	48.3 (12.5)

VAS—visual analogue scale; pre-op—pre-operative; post-op—post-operative, diff—difference; ( )—SD.

**Table 4:** Patient-reported outcomes measures (function and QOL) by the included studies.

Study	ODI			RDQ			SF-36 PCS			SF-36 MCS			SF-12 PCS	SF-12 MCS
	Pre-op	Post-op	Diff	Pre-op	Post-op	Diff	Pre-op	Post-op	Diff	Pre-op	Post-op	Diff	Diff	Diff
Ahmadian et al., 2013	50.4	30.9	19.5	-	-	-	-	-	-	-	-	-	-	-
Campbell et al., 2018	49.1	23.1	53.0	-	-	-	-	-	-	-	-	-	5.4	4.7
Khajavi et al., 2015	43	21	51.0	-	-	-	31.2	43.6	40%	43.8	52	19%	-	-
Marchi et al., 2012	66	30	36	-	-	-	-	-	-	-	-	-	-	-
Ohba et al., 2017	21.2 (6.9)	9.2 (7.4)	12	13.9 (5.5)	8.2 (5.4)	5.7	-	-	-	-	-	-	-	-
Pawar et al., 2015	-	-	19.5	-	-	-	-	-	-	-	-	-	10.3	7.4
Sato et al., 2017	50 (16)	16 (8)	34	-	-	-	-	-	-	-	-	-	-	-
Sembrano et al., 2016	43	20	23	-	-	-	37.7	61.4	62.9%	51	61.2	20%	-	-
Wu et al., 2019	59.7 (6.3)	14.8 (6.3)	44.9	-	-	-	-	-	-	-	-	-	-	-
Total	47.8 (13.3)	20.6 (7.4)	32.5 (14.9)	-	-	-	34.5 (4.6)	52.5 (12.6)	51.5 (16.2)%	47.4 (5.1)	56.6 (6.5)	19.5 (0.7)%	7.9 (3.5)	6.1 (1.9)

ODI—Oswestry Disability Index; RDQ—Roland Morris Disability Questionnaire; pre-op—pre-operative; post-op—post-operative; diff—difference; ( )—SD.

**Table 5:** Radiological outcomes by the included studies.

Study	Spondylolisthesis (% or mm)			Disc Height (mm)			Disc Angle (°)			Lumbar Lordosis (°)		
	Pre-op	Post-op	Diff	Pre-op	Post-op	Diff	Pre-op	Post-op	Diff	Pre-op	Post-op	Diff
Isaacs et al., 2016	3.4 (3.3) mm	1.8 (1.9) mm	1.7 (1.9) mm	7.6 (1.9)	9.1 (2.3)	1.5 (2.0)	9.2 (4.5)	8.5 (4.2)	−0.7	58.4 (13.4)	58.6 (13.5)	1.8 (8.0)
Khajavi et al., 2015	8.1 (20.3)%	2.5 (6.5)%	5.6%	6.6	11.3	4.7	-	-	-	-	-	-
Kono et al., 2018	5.5 (2.9)%	2.8%	2.7%	8.3	10.1	1.8	5.1	8.3	3.2	-	-	-
Marchi et al., 2012	15.1 (5.2)% {6–32}	7.1 (6)%	8.0%	-	-	55%	9.7 (3.8)	15.7 (7.1)	6.0	42.8 (15.0)	46.5 (16.2)	3.7
Pawar et al., 2015	-	-	-	L3–4: 7.4 L4–5: 8	L3–4: 13.2 L4–5: 13.3	L3–4: 5.8 L4–5: 5.3	L3–4: 11.2 L4–5: 15.6	L3–4: 12.7 L4–5: 19.6	L3–4: 1.5 L4–5: 4.0	44.1	47.5	3.4
Rodgers et al., 2012	11.1 (1.7) mm	3.6 (2.3) mm	7.5 mm	4.6 (2.2)	9.0 (2.5)	4.4	-	-	-	-	-	-
Sato et al., 2017	-	-	9%	-	-	61%	-	-	-	-	-	-
Wu et al., 2019	-	-	-	8.1 (1.7)	12.6 (1.1)	4.5	-	-	-	43.1 (12.1)	50.4 (9.4)	7.3
Total	9.6 (5.0)%	4.1 (2.6)%	6.3 (2.8)%	7.2 (1.3)	11.2 (1.9)	4.0 (1.7) (55%)	10.2 (3.8)	13.0 (4.8)	2.8 (2.5) (27.5%)	47.1 (7.6)	50.8 (5.5)	4.1 (2.3)
**Study**	**Foraminal Height (mm)**			**Foraminal Area** **Approach Side (mm^2^)**			**Foraminal Area** **Contralateral Side (mm^2^)**			**Cross-Sectional Spinal Canal Area** **(mm^2^)**		
	**Pre-op**	**Post-op**	**Diff**	**Pre-op**	**Post-op**	**Diff**	**Pre-op**	**Post-op**	**Diff**	**Pre-op**	**Post-op**	**Diff**
Isaacs et al., 2016	-	-	-	81.5	100.0	13.4 (22.7%)	89.4	101.1	7.8 (13.1%)	135.1 (62.8)	153.9 (63.0)	13.9%
Khajavi et al., 2015	19.4	23.2	19.7%	-	-	-	-	-	-	-	-	-
Kono et al., 2018	-	-	-	-	-	-	-	-	-	26.4	55.4	29%
Pawar et al., 2015	L3–4: 15.6 L4–5: 14.4	L3–4: 19.4 L4–5: 18.4	L3–4: 24.4% L4–5: 27.8%	-	-	-	-	-	-	-	-	-
Sato et al., 2017	-	-	Left: 18% Right: 16%	-	-	21%	-	-	39%	-	-	19%
Total	16.5 (2.6)	20.3 (2.5)	21.2 (4.8)%	-	-	21.9 (1.2)%	-	-	26.1 (18.3)%	-	-	20.6 (7.7)%

pre-op—pre-operative; post-op—post-operative; diff—difference; ( )—SD; { }—range.

**Table 6:** Postoperative complication.

Study	Complications	Outcome
Ahmadian et al., 2013	Anterior thigh numbness: 7 (22.5%)	100% recovery by 3 months
Campbell et al., 2018	Anterior thigh dysesthesia: 6 (33%) Hip flexion weakness: 1 (6%)	100% recovery by 2 weeks or 6 months 100% recovery by 4 weeks
Khajavi et al., 2015	Hip flexion weakness: 2 (3.3%) Anterior thigh discomfort: 3 (5%)	temporary
Kono et al., 2018	Thigh symptoms (approach side): 8 (40%) Thigh pain (contralateral side): 2 (10%)	resolved within 3 months by conservative therapy
Marchi et al., 2012	Psoas weakness: 10 (19.2%) Anterior thigh numbness: 5 (9.6%)	100% recovery by 6 weeks
Ohba et al., 2017	Thigh sensory change: 5 (10.9%) Hip flexion weakness: 4 (8.7%)	temporary
Pawar et al., 2015	Sensory deficit: 7 (18%) Anterior groin pain and thigh pain: 8 (21%) Psoas mechanical flexion deficit: 5 (13%)	100% recovery by 1 year
Sato et al., 2017	Thigh pain: 1 (5%) Thigh numbness: 1 (5%)	-
Sembrano et al., 2016	Hip flexion weakness: 9 (31.0%) Distal motor weakness (neural): 1 (3.4%; all left leg myotomes) Sensory deficit (neural): 3 (10.3%)	100% recovery by 6 or 12 months
Wu et al., 2019	Thigh pain and/or numbness: 3 (9.7%) Thigh flexion weakness: 2 (6.5%)	temporary

Number (%).

## Data Availability

Not applicable.
